# Intracochlear PLGA based implants for dexamethasone release: Challenges and solutions

**DOI:** 10.1016/j.ijpx.2019.100015

**Published:** 2019-05-21

**Authors:** E. Lehner, D. Gündel, A. Liebau, S. Plontke, K. Mäder

**Affiliations:** a Institute of Pharmacy, Martin Luther University Halle-Wittenberg, D-06120 Halle (Saale), Germany; b Department of Nuclear Medicine, Martin Luther University Halle-Wittenberg, D-06120 Halle (Saale), Germany; c Department of Otorhinolaryngology-Head and Neck Surgery, Martin Luther University Halle-Wittenberg, D-06120 Halle (Saale), Germany

**Keywords:** Biodegradable polymer, PLGA, Dexamethasone, Cochlea, Implant, Controlled release

## Abstract

The effective treatment of diseases of the inner ear is currently an unmet medical need. Local controlled drug delivery to the cochlea is challenging due to the hidden location, small volume and high sensitivity of this organ. A local intracochlear delivery of drugs would avoid the problems of intratympanic (extracochlear) drug application, but is more invasive. The requirements for such a delivery system include a small size and appropriate flexibility. The delivery device must be rigid enough for surgical handling but also flexible to avoid traumatizing cochlear structures. We developed biodegradable dexamethasone loaded PLGA extrudates for the controlled intracochlear release. In order to achieve the desired flexibility, Polyethylene glycol (PEG) was used as a plasticizer. In addition to the drug release, the extrudates were characterized in vitro by differential scanning calorimetry (DSC) and texture analysis. Simulation of the pharmacokinetics of the inner ear support the expectation that a constant perilymph drug level is obtained after few hours and retained over several weeks. Ex vivo implantation of the extrudates into a guinea pig cochlea indicate that PEG containing extrudates have the desired balance between mechanical strength and flexibility for direct implantation into the cochlea. The location of the implant was visualized by computer tomography. In summary, we postulate that intracochlear administration of drug releasing biodegradable implants is a new and promising approach to achieve local drug delivery to the cochlea for an extended time.

## Graphical abstract

**Figure:**
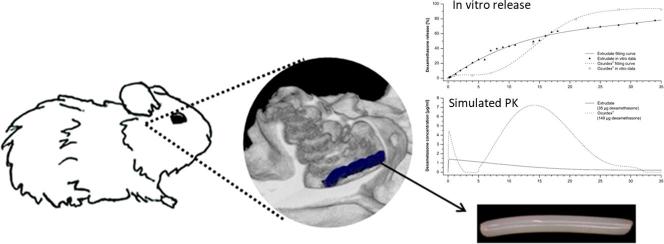

## 1 Introduction

Drug delivery to the inner ear represents currently an unmet medical need (Plontke and Salt, 2018). In many cases, insufficient drug concentrations are achieved after intravenous injections due to the presence of physiological barriers and rapid clearance (Devare et al., 2018, Plontke et al., 2007a, Plontke et al., 2007b, Salt and Plontke, 2018, Salt and Plontke, 2009). Local controlled drug delivery to the cochlea is challenging due to its location in the petrous bone, the small volume and the high sensitivity of its sensory and non-sensory structures. Advantages of local drug delivery to the inner ear include the bypassing of the blood–labyrinth barrier, the possibility to achieve higher drug concentration in the inner ear, the avoidance of “first-pass” metabolism, reduction of the overall dose and decreased systemic side effects (Plontke et al., 2017). The main administration sites for drug delivery to the inner ear in humans are extracochlear (intratympanic, IT) and intracochlear (IC) application. Extracochlear administrations are less invasive and more widely used, however, a close contact to and a long drug persistence at the round window (RW) membrane are crucial. A detailed discussion of the main drug delivery systems has been recently published by Mäder et al. (2018). Both non-degradable and biodegradable drug delivery systems have been postulated. Among the non-degradable systems, silicon based implants show great promise to improve drug delivery to the inner ear (Gehrke et al., 2019, Gehrke et al., 2016, Krenzlin et al., 2012). Our work is focused on biodegradable drug delivery systems. In a previous clinical pilot study we placed a Ozurdex® drug delivery implant into the RW niche and onto the RW membrane as a salvage therapy for idiopathic sudden sensorial hearing loss (ISSHL) (Plontke et al., 2014). Ozurdex® is a PLGA based drug delivery system that can elute dexamethasone over several weeks (Bhagat et al., 2014). The approved use of Ozurdex® is the intravitreal injection to provide sustained delivery of dexamethasone to the eye (Allergan, 2014). Indications include the treatment of macular edema and non-infectious inflammation of the uvea (uveitis) affecting the posterior segment of the eye. In our study, we were able to show that the local administration of Ozurdex® implants to the RW showed encouraging results for the treatment of sudden hearing loss (Plontke et al., 2014). However, it was necessary to adapt the size of the clinically used Ozurdex® implant in order to place it into the RW niche. We observed that “pieces of the rigid implants may show sharp edges at their breaking points” (Plontke et al., 2014). Thus, it became clear, that the adaptation of a clinically used system for the eye represents a good starting point, but not the ideal system for a controlled drug delivery to the inner ear. Therefore, key properties of the implant (e.g. size, shape, mechanical strength) should be designed specifically to the requirements of the ear.

Intracochlear delivery avoids the main problems of intratympanic drug application (rapid clearance from the middle ear, limited permeability through the RWM, high variability in inner ear drug concentrations). It is more efficient than intratympanic delivery but also more invasive (Hahn et al., 2012). This drawback of invasiveness and its associated risks to the inner decreases, if combined with a procedure that requires an opening of the cochlea, e.g. in case of insertion of a cochlear implant (CI) electrode carrier (Plontke et al., 2017). So far, intracochlear drug delivery with controlled release characteristics is mainly linked to drug eluting electrodes of cochlear implants (Astolfi et al., 2014, Bas et al., 2016, Liu et al., 2015, Wilk et al., 2016). These include limited possibilities for personalized treatment due to a fixed combination of the cochlear implant and drug containing material and limited drug load. In addition, the approval of a drug device combination appears more challenging then for an intracochlear controlled release device only. Thus, the development of intracochlear drug loaded biodegradable implants which might be administrated with or without a cochlear implant is highly desirable because it gives more opportunities to personalized medicines and permits an independent optimisation of both systems. In a clinical pilot study including two patients with cochlear implants we placed two short pieces (<3 mm in lengths) of the Ozurdex® implant in parallel to the cochlear implant in the basal region of the cochlear to treat a developing local inflammation (Plontke et al., 2017). In this study it was demonstrated that there is enough space in the scala tympani to place a drug delivery system besides a cochlear implant. However, the pieces of the Ozurdex® implant sometimes have broken during insertion because of their stiffness. As mentioned before the pieces may show sharp edges and could harm the cochlear wall.

It was therefore the aim of this study to develop a PLGA based biodegradable implant for intracochlear delivery of drugs with appropriate size and mechanical properties and to prove the general feasibility of its administration. Dexamethasone was selected as drug, because it has shown a protective effect against electrode insertion trauma and to prevent fibrotic tissue formation around the cochlea implant electrode carrier (Bas et al., 2016, Hahn et al., 2012, Jia et al., 2016, Wilk et al., 2016). The eye implant Ozurdex® (thickness 460 µm) served us as a starting point. However, Ozurdex® itself is not suitable for intracochlear administration because of its stiffness. In order to test the delivery system in guinea pigs, a widely used animal model for hearing disorders and studies on inner ear pharmacokinetic, we selected 300 µm as a suitable thickness for the polymer implant based on the anatomical characteristics of the guinea pig cochlea.

In addition, the release profile of Ozurdex® would not be optimal for the application on prevention of insertion trauma, because a lag time over several days has been described (Bhagat et al., 2014). A lag time is not desirable, because a fast drug release is essential to dampen the initial inflammation phase which is initiated within few hours after implantation (Jia et al., 2016, Takumi et al., 2014). Due to the fast elimination rate of glucocorticoids in the inner ear fluids, an additional application during the implantation surgery can only bridge a short time period (Plontke et al., 2007a, Plontke et al., 2007b, Salt et al., 2012). Nevertheless, in order to get therapeutic inner ear drug concentrations immediately after insertion of the CI electrode such a procedure may still be useful in combination with the use of an intracochlear drug delivery device.

PEG was selected as a plasticizer in the PLGA based implant. We expected that the use of PEG might provide the implant with the desired sufficient mechanical properties and overcome the undesired lag time, because it will accelerate wetting and water penetration into the implant. Melt extrusion was used for manufacturing the implants. The implants were characterized by light microscopy and differential scanning calorimetry. Texture analysis was used to measure the mechanical properties. In addition, in vitro drug release studies were conducted. The delivery device was tested for general feasibility of implantation *ex vivo* in a guinea pig cochlea. To monitor the location of the implant within the inner ear, CT images of BaSO_4_-loaded implants were recorded.

## 2 Materials and methods

### 2.1 Materials

Poly-(d,l-lactic-co-glycolic acid) (PLGA, Expansorb® 50-2A, Mw = 10.4 kDa measured by GPC) was provided by Merck KGaA (Darmstadt, Germany). Dexamethasone was bought from Caesar & Loretz GmbH (Hilden, Germany). Polyethylene glycol (PEG) 1500 g mol^−1^ was purchased from Alfa Aesar (Haverhill, USA). Barium sulfate nanoparticles (D90 = 0.35 µm) (Blanc Fixe® Solvay, Massa, Italia) were used as contrast agent for implants monitored by computer tomography (CT). Testing media selected was Dulbecco’s Phosphate Buffered Saline (8 g/L NaCl, 0.2 g/L KCl, 1.15 g/L Na_2_HPO_4_, 0.2 g/L KH_2_PO_4_), adjusted to pH 7.4. To avoid microbial growth, sodium azide 0.02% was added to the phosphate buffer. Acetonitrile (VWR-International, Darmstadt, Germany) and double distilled water were used for the HPLC method.

### 2.2 Implant preparation

For implant preparation, two different ratios of PLGA, PEG 1500 and dexamethasone were used (Table 1). The mixtures were pulverized with a cryomill (Retsch GmbH, Haan, Germany). The following settings were used: cycles 4, precool time 2 min, run time 1.5 min, cool time 1 min, rate 15 CPS. The grinding jar was continually cooled with liquid nitrogen. The powder was removed after room temperature was reached.

**Table 1:** Composition of Formulations.

Formulation	PLGA [% m/m]	PEG 1500 [% m/m]	Dexamethasone [%m/m]	BaSO_4_ [% m/m]
A	90	–	10	–
B	80	10	10	–
C	75	15	10	–
D	80	10	–	10

The solid implants were formed by hot-melt extrusion (ZE 5 ECO; Three-Tec GmbH; Seon; Swiss) with an extrusion screw frequency of 60 rpm. The three heating zones were set to 50, 50 and 52 °C. A plate with a definite gap with 0.3 mm diameter was put on the end of the extrusion tool. The homogenized mixture was fed to the nitrogen air cooled barrel. The extruded material was collected and stored in a fridge at 2–8 °C.

### 2.3 Optical light microscope

For the visualization of the prepared extrudates two different microscopes were used. Size and swelling behavior of PLGA implants were studied using an Olympus SZX9 reflected-light microscope.

Samples (n = 3) were prepared by cutting the extrudates under the microscope to a length of 3.0 mm. The swelling of the extrudates was monitored using 1 mL glass vials filled with 1 mL phosphate buffer pH 7.4. The vials were kept at 37 °C in a shaker with light protection (Memmert GmbH + Co. KG, Schwabach, Germany). The extrudates were carefully removed at pre-determined time points and examined microscopically. Pictures were taken with an UC30 camera (Olympus Optical Co., Hamburg, Germany) and the dimensions of the implants were analyzed with OLYMPUS stream motion (Olympus Optical Co., Hamburg, Germany).

The presence of dexamethasone crystals was examined with an Axiolab transmitted-light microscope with polarization filter unit (Carl Zeiss MicroImaging GmbH, Göttingen, Germany). For this purpose, the extrudates were cut in 30 µm slices using a Leica RM 2245 microtome (Leica Biosystems, Nussloch, Germany).

### 2.4 Differential scanning calorimetry (DSC)

DSC measurements for PLGA, PEG 1500 and the extrudates (immediately after preparation) were recorded with a Mettler Toledo DSC 823e module (Mettler Toledo, Gießen, Germany) in standard aluminum sample pans. Every sample was cooled down to −20 °C and kept at this temperature for 20 min. The sample was then heated up to 60 °C with a heating rate of 5 K/min. Data recording and processing of first heating cycles were carried out with the software STARe V15.00 (Mettler Toledo, Gießen, Germany).

### 2.5 Mechanical properties

Using the Texture Analyzer (CT3-4500, Brookfield-Rheotec, Germany), the required force to push a metal blade with a constant velocity into the extrudates was measured. Therefore, the samples were placed onto a stage (accessory TA-RT-KIT, Brookfield-Rheotec, Germany). Experiments were conducted at 20 °C using the deformation mode with a blade (accessory TA7, knife edge, Brookfield-Rheotec, Germany) and a scan velocity of 0.01 mm/s.

The trigger force was adjusted to 0.02 N. The test ended after a penetration distance of 0.2 mm. Data recording and processing were carried out with the software TexturePro CT V1.4 Build 17 (Brookfield-Rheotec, Germany).

### 2.6 Determination of drug incorporation efficiency

One mg of the extrudate was dissolved in 100 µL acetone and filled up to 1 mL with acetonitrile. Dexamethasone in the solution was measured by the HPLC method described below. The individual values for three replicate determinations and their mean values are reported. Drug loading was calculated as follows:

$$DrugLoading\left(\%\right)=\frac{\mathrm{massofdruginextrudate}}{\mathrm{massofextrudate}}\times 100$$

### 2.7 *In vitro* dexamethasone release

One mg of extrudates was placed in 1 mL glass vials filled with 1 mL phosphate buffer pH 7.4 and the vials were slightly agitated in a shaker with light protection (Memmert GmbH + Co. KG, Schwabach, Germany) at 37 °C.

Total buffer volume was withdrawn with a Hamilton syringe at regular time intervals and analyzed according to the described HPLC method. Appropriate volume of fresh phosphate buffer was replaced after taking samples. Each experiment was conducted in triplicate.

### 2.8 High performance liquid chromatography

A modified method from United States Pharmacopeia was used to quantify the amount of dexamethasone which has been released. A Jasco high-performance liquid chromatography (HPLC) system with a PU-1580 Pump equipped with LG-1580-04 quaternary gradient unit, AS 1559 Intelligent Auto Sampler, UV 1559 intelligent UV/VIS Detector (all Jasco, Oklahoma City, USA). Purospher® Star RP-18 endcapped (5 µm) column (Merck Millipore, Billerica, Massachusetts, USA), operated at 40 °C, was used. Acetonitrile and double distilled water in a ratio 30:70 V/V were used as the mobile phase at a flow of 1 mL min^−1^. 20 µL of the sample were injected and analyzed at λ = 258 nm. The retention time for dexamethasone was found to be 8 min. Data recording and processing were carried out with the software ChromNAV Ver.2 (Jasco, Oklahoma City, USA).

### 2.9 Simulation of achieved inner ear drug levels

For estimation the inner ear drug levels (i.e. dexamethasone concentration) by the implants in a clinical setting we used a validated computer model based on a finite element algorithm (developed by A. Salt) of drug distribution in the inner ear fluids that take into account movement and elimination processes of substances in the cochlea ducts (Washington University Cochlear Fluids Simulator, Version 3.23, (Salt, 2002, Plontke et al., 2007b). The virtual model of the cochlear ducts used in this program based on 3D reconstruction of a segmented human inner specimen obtained by nondestructive micro computed tomography and orthogonal-plane fluorescence optical sectioning microscopy (Skinner et al., 2007). The time course of dexamethasone concentrations in the scala tympani perilymph of the human inner ear after implantation the drug delivering devices into the basal part of scala tympani was simulated over 5 weeks. The simulations based on in vitro release data of PLGA extrudates of Formulation B and the Ozurdex® implant are shown in Fig. 5. The dimensions of the PLGA extrudate for simulation were set to 300 µm in diameter and 3 mm in length and the total drug amount to 35 µg dexamethasone. Dimensions of the Ozurdex® implant used for simulation were either set to 146 µm in diameter and 3 mm in length so that the drug load is equal to the drug amount in the PLGA extrudate of 35 µg or set equal to the dimensions of the PLGA extrudate. The respective drug amount in such a piece of Ozurdex® would be 149 µg dexamethasone in relation to the total drug load in the Ozurdex® implant of 700 µg dexamethasone (460 µm in diameter and 6 mm in length).

### 2.10 *Ex vivo* implantation into a guinea pig cochlea

For CT scanning, barium sulfate loaded implants were prepared and implanted into the scala tympani of a guinea pig cochlea of a freshly scarified animal by insertion through a cochleostomy. After scarifying the animal by intracardiac injection of KCl under deep anesthesia the bulla bone was isolated and the cochlea was prepared and rinsed in isotonic PBS buffer. The basal turn of the cochlea near the RW was minimal opened with a diamond drill (0.4 mm). A 0.35 mm × 3 mm implant was inserted slowly into the scala tympani through the cochleostomy. The cochlea was again stored in isotonic PBS buffer.

### 2.11 Computer tomography scan

Imaging of the implant inserted into a guinea pig cochlea was performed with an animal CT scanner with 720 projections and an X-Ray energy of 70 kVp using a nanoScan PET/CT (Mediso GmbH, Münster, Germany). For the reconstruction (voxel size: 25 µm × 25 µm × 25 µm, filter: Cosine) of CT images the Nucline Software (Mediso GmbH, Münster, Germany) was used. The reconstructed images where analyzed with π.pmod Software (PMOD Technologies LLC, Zürich, Switzerland), depicting VOI of implants by a grey value intensity based threshold algorithm.

## 3 Results and discussion

### 3.1 Macroscopic characterization

**Fig. 1:**
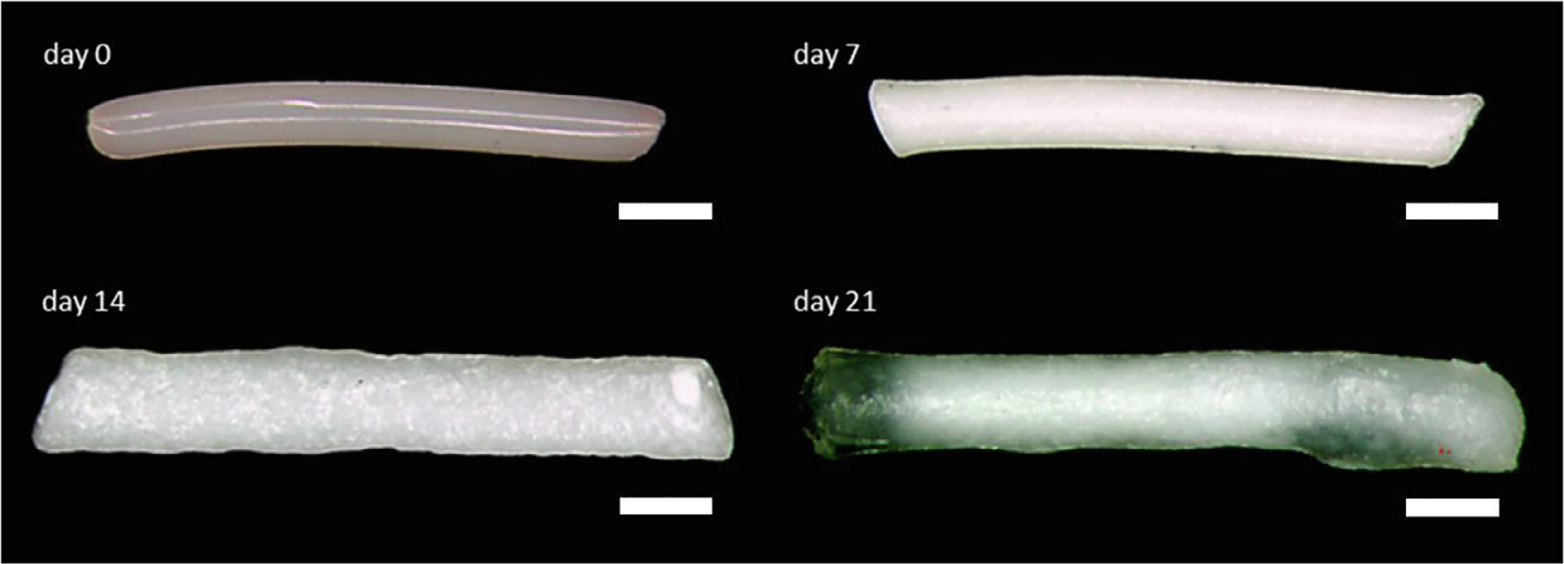
Morphology of Formulation B implant prepared by hot-melt extrusion at day 0 and after incubation in PBS at day 7, day 14 and day 21; scale bar indicates 500 µm.

The optical properties and the swelling behavior were studied by macroscopic observations. In the case of biodegradable and expandable implant systems, the effect of swelling on the diffusivity of the drug are important parameters that influence the release profile. However, all formulations were visually turbid and rod shaped after preparation by hot-melt extrusion at 50 °C. Especially Formulation D showed an opalescence which is a proof for the small size of the BaSO_4_ nanoparticles. Fig. 1 shows Formulation B as an example. The fact that the diameter of the extrudate (350 µm) exceeds the diameter of the extruder gap (300 µm) indicates the viscoelastic properties of the polymer. After exposure to buffer, the dimensions of the implant increase (Table 2). The implants show a time dependent swelling. The swelling was not isometric, but more pronounced in thickness compared to the length. The largest expansion (620 µm) in width was observed after three weeks. After four weeks, the implants disintegrated into several fragments. Such fragmentation has also been reported in clinical cases for the Ozurdex® implant (Roy and Hedge, 2013). The time dependent changes of the dimensions for all 3 implants of Formulation B were illustrated in Figs. A.1 and A.2.

**Table 2:** Time dependent swelling of Formulation B.

Day	Length [µm]	Length increase [%]	Width [µm]	Width increase [%]
0	3140		350	
7	3350	6.7	395	12.9
14	4430	41.1	560	60.0
21	4500	43.3	620	77.1

### 3.2 Microscopic characterization

**Fig. 2:**
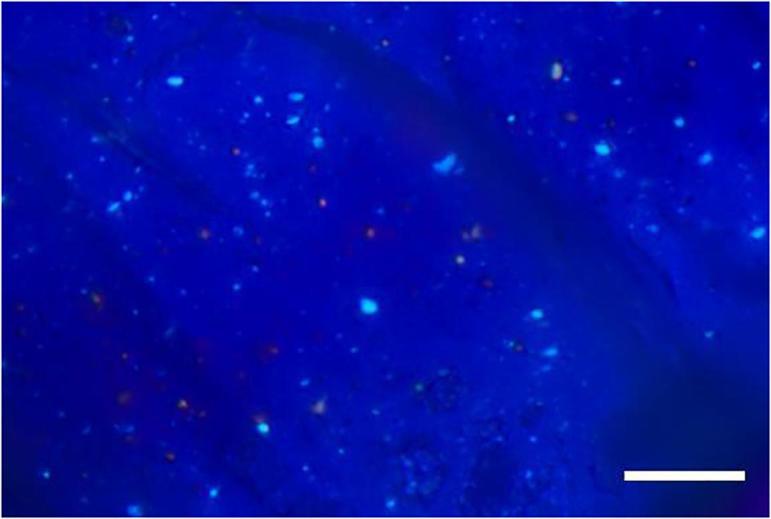
Slice of a Formulation B extrudate in a 500 fold magnification under polarized light; scale bar indicates 20 µm.

With the transmitted-light microscope, the presence of dexamethasone crystals and the particle size were determined. The 30 µm extrudate slice of Formulation B showed crystals with an average diameter of about 2 μm. The crystals were homogeneously distributed and showed approximately the same size (Fig. 2). Even with a dexamethasone load of 1%, crystals could be detected in previous experiments using a film method.

### 3.3 Differential scanning calorimetry

**Fig. 3:**
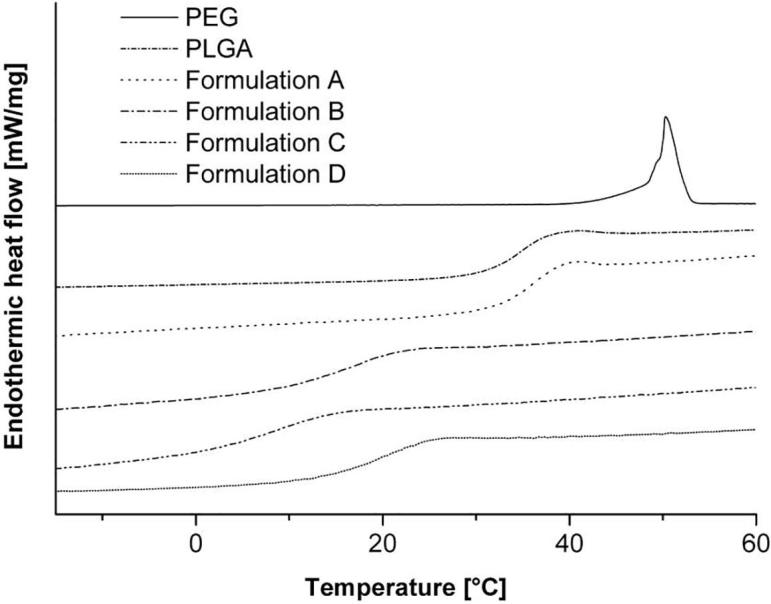
Thermograms of PLGA, PEG and extrudates. Measurements were carried out with a heating rate of 5 K/min between −20 and 60 °C.

Thermal properties of the extrudates and the ingredients were determined by differential scanning calorimetry. The used PLGA is an amorphous solid with a glass transition (Tg) at 35 °C. Dexamethasone is a crystalline drug which had a melting point at 262 °C (not shown). The presence of crystalline dexamethasone in the different formulations could not be detected by DSC because of the degradation temperature of PLGA at 270 °C (Fredenberg et al., 2011). PEG is also crystalline and shows a melting point at 51 °C (Fig. 3). Formulation A had nearly the same Tg as PLGA, hence dexamethasone has no influence on the Tg. All formulations with PEG (B, C, D) showed a lower Tg than pure PLGA and no melting point at 51 °C for PEG. In addition, the Tg is below room temperature and therefore, the implants are in the rubbery state during application. The Tg is decreasing with a higher amount of PEG. Therefore, PEG is a good plasticizer to lower the glass transition of PLGA extrudates. A summary of all glass transition temperatures is shown in Table 3.

**Table 3:** Glass transition and melting temperatures of single components (PLGA, PEG, dexamethasone) and hot-melt extrudates.

Substance	T_g_ [°C]	T_m_ [°C]
PLGA	35	–
PEG 1500	–	51
Dexamethasone	–	262
Formulation A	36	–
Formulation B	17	–
Formulation C	9	–
Formulation D	19	–

### 3.4 Texture analysis

**Fig. 4:**
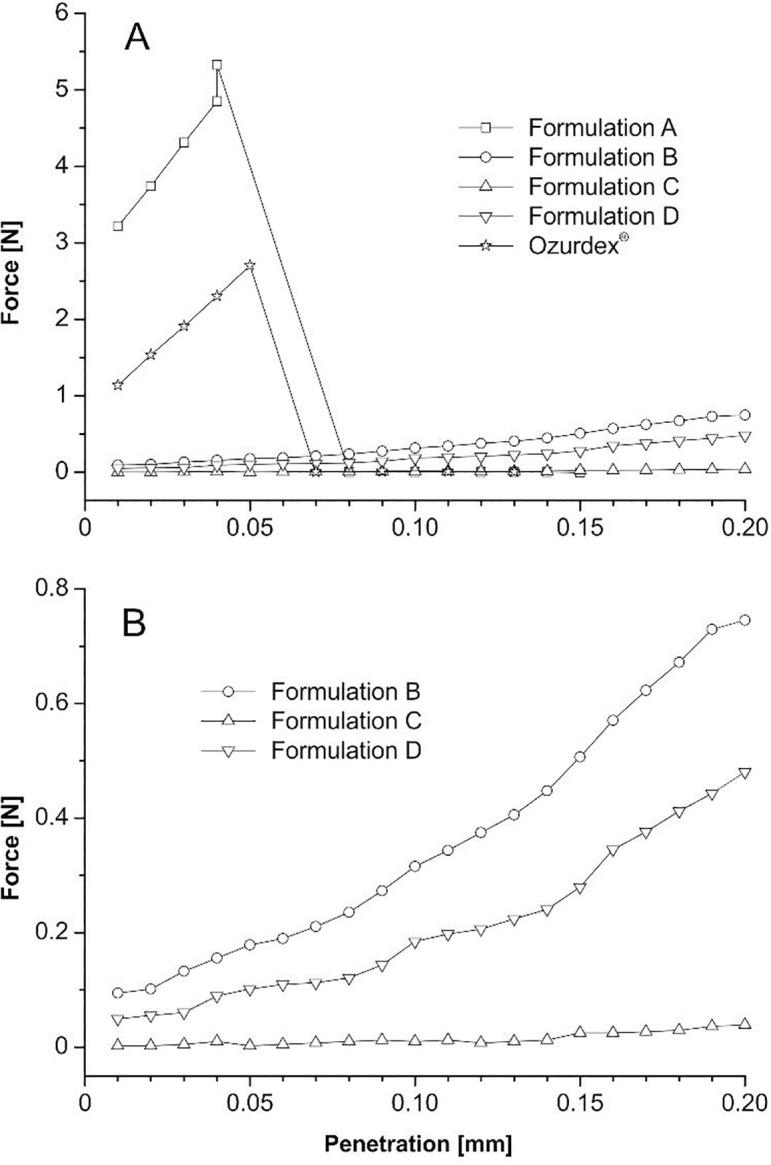
Tensile test profile of the extrudates (A); enlarged scaling for f better visualization (B).

For an application to the inner ear, it is important to have an implant which combines flexibility with sufficient mechanical stability. If the flexibility is too high, the implant cannot be easily inserted through the RW membrane into the cochlea. Injuring of the inner ear can be a big problem if too rigid implants are used. The mechanical properties of the implants were studied by texture analysis. A small blade was forced to penetrate over a distance of 0.2 mm into the extrudates and the resulting force was measured. Ozurdex® and the PEG-free Formulation A showed a fast increase of the force, indicating a high mechanical resistance (Fig. 4). After a distance of about 0.04 mm the extrudates cracked in two pieces and a maximal force for Formulation A was found at 5.33 N and 2.70 N for Ozurdex®. In contrast, for PEG containing implants, the blade penetrated the whole distance over 0.2 mm without cracking the extrudates. A maximal force for Formulation B was found at 0.75 N. It is clear to see that Formulation C was softer than Formulation B. For Formulation C, nearly no rise in force was detected (F_max_ = 0.037 N). In summary, PEG softens the extrudates and prevents cracking. The impact of PEG on the mechanical properties is strong, as only 14.1% and 0.7% of the mechanical strength of Formulation A were observed for polymers containing 10% and 15% PEG respectively. For the CT studies, the barium sulfate loaded extrudates should not differ from the drug loaded extrudates. The force/displacement curve of Formulation D showed a similar behavior to Formulation B and C. No cracking was observed and the maximum force (Fmax = 0.48 N) was in the same range.

### 3.5 Drug incorporation efficiency

The theoretical drug load for the polymer extrudates was 10%. Experimentally determined values indicated drug loads of 10.37% ± 0.18% (Formulation A), 10.71% ± 0.14% (Formulation B) and 10.85% ± 0.21% (Formulation C). Thus, it can be stated that the manufacturing process led to reproducible implants with a drug load of about 10% as expected.

### 3.6 *In vitro* dexamethasone release

**Fig. 5:**
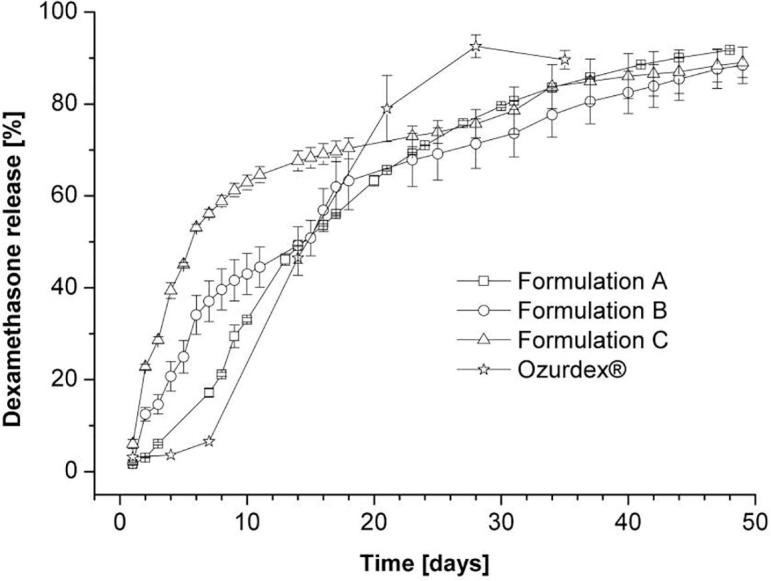
Cumulative release of dexamethasone from PLGA extrudates in phosphate buffer pH 7.4 and Ozurdex®; n = 3; the error bars indicate the standard deviation.

The release of dexamethasone from the PLGA extrudates has been investigated at 37 °C in phosphate buffer pH 7.4. Fig. 5 shows the dexamethasone release kinetics. Only a small amount of the incorporated dexamethasone (6.5%) is released from Ozurdex® (Bhagat et al., 2014) within the first week. Thereafter, the release accelerates and reaches almost 50% after two weeks and 80% after three weeks. Release profiles with this pattern are quite commonly observed for PLGA polymers and connected to initial slow water penetration and the autocatalytic degradation of the polymer. A detailed discussion of the main parameters and the drug release mechanisms from PLGA polymers has been published by Fredenberg et al. (2011). The low amount of initial drug release is in conflict with the desired kinetic profile, which aims for no lag time and an initial equal or even slightly higher release rate. Compared to Ozurdex®, higher initial release rates were obtained with all developed implants. After one week, 17.2%; 37.0% and 56.2% were released from Formulations A, B, C respectively. However, the PEG free Formulation A showed only minor drug release (6%) in the first three days, compared to 14.6% and 28.6% drug release of Formulations B and C. Therefore, the release profiles of Formulations B and C match much better with the desired kinetics. It can be concluded, that the incorporation of PEG (10% or 15%) has a beneficial effect of overcoming the undesired slow initial release. The initial release is tunable by the amount of the incorporated PEG.

Despite the higher initial release of Formulations B and C, a longer release time is observed which is due to the lack of the sigmoidal shape of the release profile. Most likely, the incorporation of PEG is not only accelerating the water penetration, but also preventing (at least partially) the autocatalytic degradation of the polymer by increasing the diffusivity and release of hydrophilic acidic degradation products (e.g. lactic and glycolic acid).

### 3.7 Simulation of inner ear drug levels

**Fig. 6:**
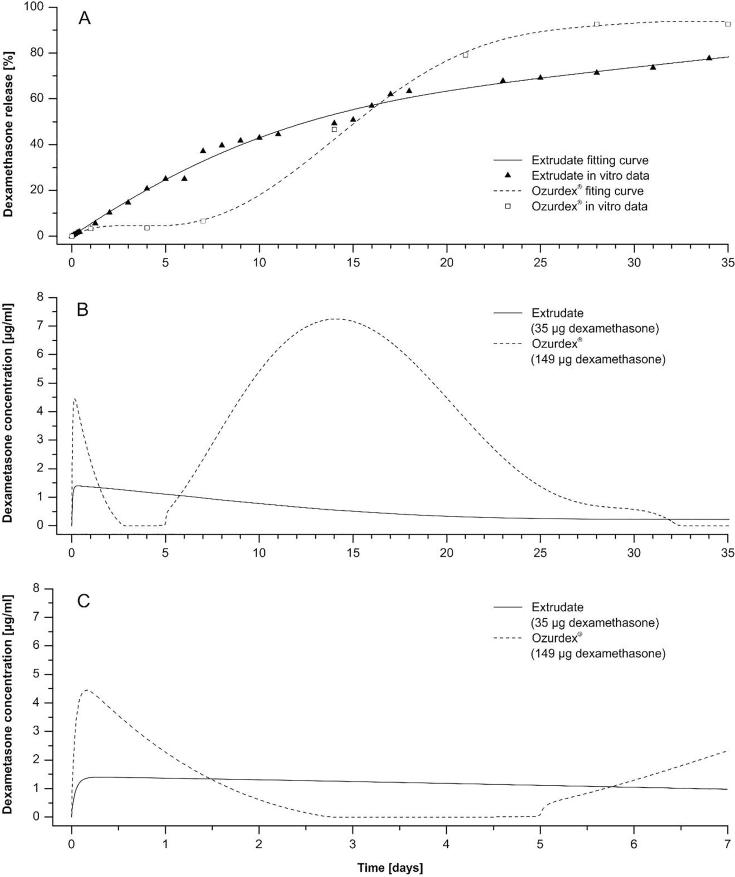
Simulation of the inner ear drug levels after implantation the PLGA extrudate (Formulation B) and Ozurdex® into the human inner ear. (A) Fitting curves based on in vitro relative cumulative release of dexamethasone from PLGA extrudates and Ozurdex® (see Fig. 5) used for simulation. (B, C) Time course of the simulated inner ear dexamethasone levels in the perilymph of scala tympani in the human inner ear for the PLGA extrudate and Ozurdex® over 5 weeks (B) and during the first week after implantation (C). Total drug amount in the PLGA extrudate was 35 µg dexamethasone whereas drug load in the Ozurdex® implant with equal dimensions as the PLGA extrudate was set to 149 µg dexamethasone.

Simulations of the complex pharmacokinetics of the inner ear is a powerful method to understand the impact of different parameters (e.g. drug properties, release rate, dimensions of the delivery system) on the time dependent distribution of drugs in the inner ear. To estimate the inner ear drug levels by using the PLGA extrudates in a clinical setting we simulated dexamethasone concentrations of cochlear PLGA implants localized into the basal part of scala tympani in the human inner ear. Formulation B was selected for the simulation since the in vitro drug release of this type shows a constant release kinetic and no lag phase. We also simulated the inner ear drug levels using a piece of Ozurdex® that has the same dimensions as the extrudate to compare the impact of both the different release kinetics and the differing possibilities for maximum drug loading of the PLGA extrudate and Ozurdex® on achieved drug levels (Fig. 6A). Please note that in consequence the amounts of dexamethasone are quite different: The simulated implants contain 149 µg drug (Ozurdex®) or 35 µg (Formulation B). Therefore, the simulations show the respective maximum achievable drug levels for both drug depots.

The simulations of the inner ear drug levels show a multiphase behavior for the Ozurdex® implant. A first maximum is rapidly reached within few hours (Fig. 6C). However, after about 6 h, the simulated dexamethasone concentrations in the perilymph of the scala tympani decrease and almost zero concentrations are obtained for the time between 2.5 days and 5 days. Thereafter, the simulated concentrations are increasing again to reach a peak after two weeks. In the following time, the simulated concentrations decline again to reach the baseline after one month. In contrast to the simulated piece of the Ozurdex® implant, the predicted drug concentrations in the inner ear released from the Formulation B lack the high fluctuations. The simulation predicts that the maximum will be reached already after few hours. Thereafter, an almost constant and very long lasting drug level is predicted by the simulation (Fig. 6B and C).

**Fig. 7:**
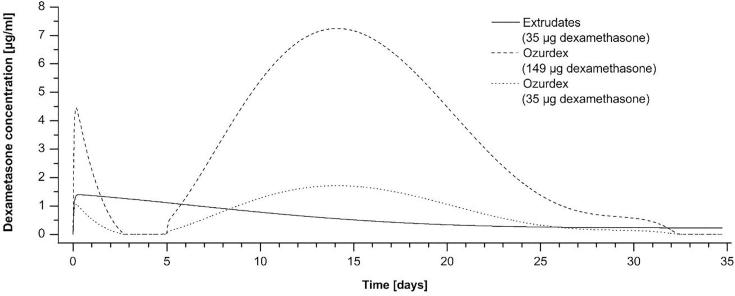
Simulation of the time course of inner ear dexamethasone levels in the perilymph of scala tympani in the human inner ear after implantation the PLGA extrudate (Formulation B) and two pieces of Ozurdex® with different drug load. Simulated time course of drug concentrations for the extrudate and an Ozurdex® piece containing 149 µg dexamethasone was taken from Fig. 6. In addition, the time course of inner ear drug levels was simulated for a smaller Ozurdex® piece containing 35 µg dexamethasone. Overall drug levels achieved by the Ozurdex® piece containing 35 µg dexamethasone are below toxic drug concentrations but the time periods of insufficient drug levels are extended.

The simulations illustrate how different release kinetics are expected to translate into different drug concentrations in the inner ear. The initial higher concentration which is predicted for the Ozurdex® piece results from the 4.2 times higher drug content despite an initially lower percentage based release rate (Fig. 6A). The high fluctuations for the Ozurdex® piece are expected to lead to insufficient drug concentrations (days 2–5) and overdosing (days 10–20). It was shown that dexamethasone concentrations above 1.2 µg/ml starts to have toxic effects on outer and inner hair cells after 5 days incubation (Jia et al., 2016). It would be possible to lower the achieved drug levels by the Ozurdex® piece below this critical concentration using a smaller piece with a lower total drug load. However, this would elongate the time of insufficient drug concentrations during the first week and shorten the overall time of drug release (Fig. 7).

Especially the drug free time period during the first week for the Ozurdex® piece is of major concern since inflammation processes after CI implantation starts within few hours and main inflammation phase occurs during the first week of implantation (Astolfi et al., 2014). Intracochlear drug delivering devices based on not absorbable silicone have already been tested in the CI insertion trauma model in guinea pigs. It was shown that drug depots loaded with 20.4 µg dexamethasone protected hearing thresholds and outer hair cell function in implanted animals and lowered the expression of the inflammation marker TNF-a in cochlear tissue (Liu et al., 2015). Another study using the same type of drug devices could show less infiltration of the cochlea with lymphocytes, macrophages, and giant cells in animals implanted with the dexamethasone eluting drug depot (Farhadi et al., 2013). A pharmacokinetic study revealed that these silicone based drug delivering devices achieved a steady state drug concentration about 0.1 µg/ml one week after implantation in the scala tympani (Liu et al., 2015). The simulations show that formulation B can cover this concentration range by reaching a steady state concentration between 0.5 and 1 µg/ml dexamethasone when using the maximum possible drug loading of 35 µg. The results of the simulations underline the high potential of Formulation B, because they predict almost constant drug levels in the inner ear over several weeks and drug levels can be adjusted to the therapeutic range by dosing the total drug load.

### 3.8 *Ex vivo* implantation and CT measurements

**Fig. 8:**
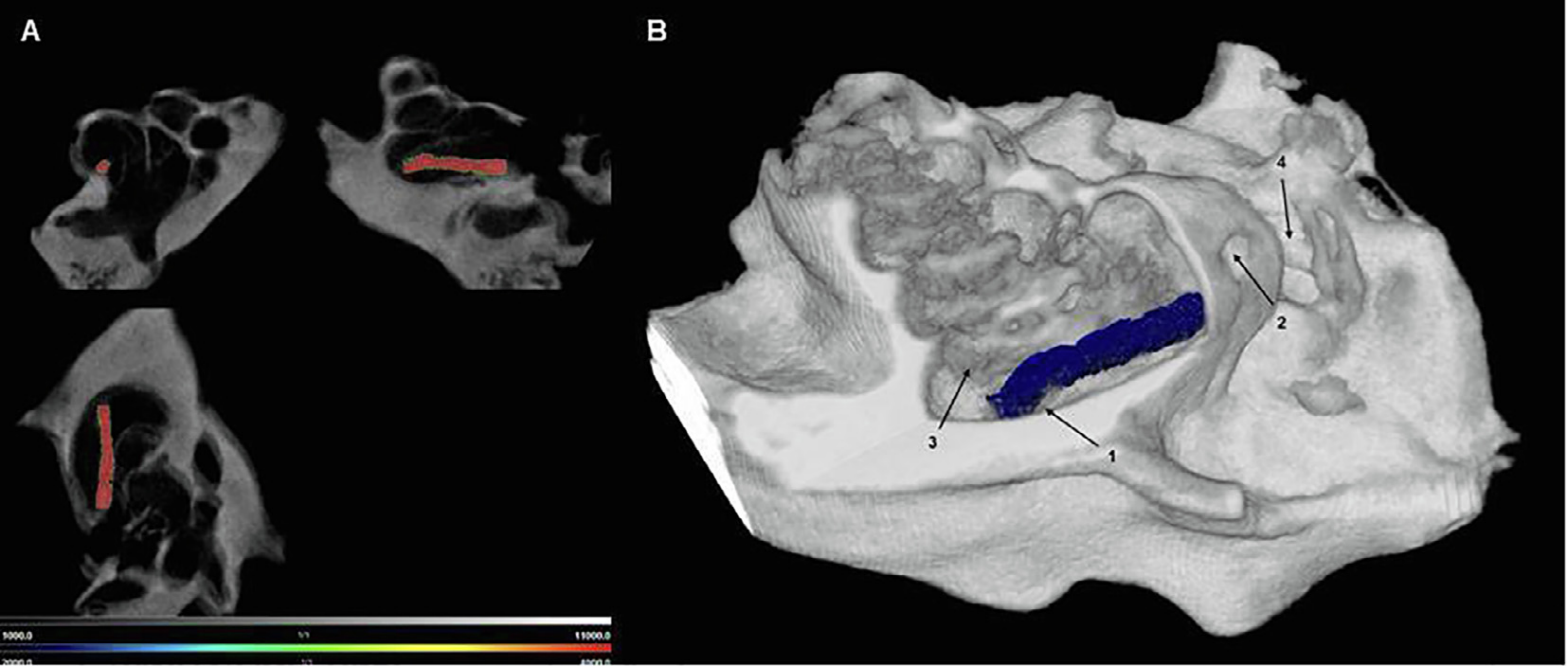
Ex vivo CT-Images of guinea pig cochlea with a Formulation D implant; (A) axial slices of the cochlea with the highlighted implant in red; (B) 3D reconstruction with the Nucline Software; 1 = implant with bended end; 2 = cochleostomy; 3 = basilar membrane; 4 = RW niche. (For interpretation of the references to colour in this figure legend, the reader is referred to the web version of this article.)

BaSO_4_ loaded polymer extrudates (Formulation D) were prepared and implanted into the scala tympani of a guinea pig cochlea by insertion through a cochleostomy. The basal turn of the cochlea near the RW was minimal opened with a diamond drill (0.4 mm). A 0.3 mm × 3 mm implant was inserted slowly into the scala tympani through the cochleostomy. The implant could be inserted without subjective resistance. No problems were notified during the insertion procedure due to the desired mechanical properties of the implant which combine flexibility with sufficient mechanical stability. After intracochlear implantation into the scala tympani of a guinea pig cochlea the position of the implant was examined by CT. The CT scans showed no macroscopic traumatization of any inner ear structures. Axial slice data clearly show the location of the implant in the scala tympani (Fig. 8A). A concentration of 10% BaSO_4_ was sufficient to visualize the small implant in the cochlea. Fig. 8B shows the 3D reconstruction of the intact implant lying in the scala tympani of the basal turn. The implant has slightly bent and it seems to touch the basilar membrane, which delimits the scala tympani from the scala media. In future studies, implants with a length of 2.0 mm will be used to avoid any physical stress on the basilar membrane.

## 4 Conclusion

Very thin (about 350 µm diameter) rod shaped dexamethasone loaded PLGA based implants with a drug load of 10% were prepared by hot-melt extrusion at 50 °C. The use of PEG as an additional excipient was beneficial in two aspects: (i) PEG softens the extrudates and prevents the cracking during bending. An adequate flexibility for intracochlear administration was reached and confirmed by texture analysis and *ex vivo* implantation in an animal model. (ii) In addition, PEG accelerated the initial drug release rate. Therefore, in vitro release kinetics displayed no significant lag time. Overall, the release kinetics did match the desired profile with a controlled release over several weeks. The simulations of the pharmacokinetics in the human ear predict that within few hours a constant drug level will be achieved in the perilymph of the scala tympani. The constant drug concentration is expected to last for several weeks. The study shows the general suitability of biodegradable intracochlear drug delivery systems. The developed intracochlear drug loaded implant might be administrated with or without a neuroprosthetic cochlear implant. It gives more opportunities to personalized medicines and permits an independent optimisation of both systems.

## Declaration of Competing Interest

The authors declare that they have no known competing financial interests.

## References

[b0005] Allergan (2014). OZURDEX® (dexamethasone intravitreal implant) for intravitreal injection. Packag. Inser..

[b0010] Astolfi L., Guaran V., Marchetti N., Olivetto E., Simoni E., Cavazzini A., Jolly C., Martini A. (2014). Cochlear implants and drug delivery: in vitro evaluation of dexamethasone release. J. Biomed. Mater. Res. – Part B: Appl. Biomater..

[b0015] Bas E., Bohorquez J., Goncalves S., Perez E., Dinh C.T., Garnham C., Hessler R., Eshraghi A.A., Van de Water T.R. (2016). Electrode array-eluted dexamethasone protects against electrode insertion trauma induced hearing and hair cell losses, damage to neural elements, increases in impedance and fibrosis: a dose response study. Hear. Res..

[b0020] Bhagat R., Zhang J., Farooq S., Li X.-Y. (2014). Comparison of the release profile and pharmacokinetics of intact and fragmented dexamethasone intravitreal implants in rabbit eyes. J. Ocul. Pharmacol. Ther..

[b0025] Devare J., Gubbels S., Raphael Y. (2018). Outlook and future of inner ear therapy. Hear. Res..

[b0030] Farhadi M., Jalessi M., Salehian P., Ghavi F.F., Emamjomeh H., Mirzadeh H., Imani M., Jolly C. (2013). Dexamethasone eluting cochlear implant: histological study in animal model. Cochlear Implants Int..

[b0035] Fredenberg S., Wahlgren M., Reslow M., Axelsson A. (2011). The mechanisms of drug release in poly(lactic-co-glycolic acid)-based drug delivery systems – a review. Int. J. Pharm..

[b0040] Gehrke M., Sircoglou J., Gnansia D., Tourrel G., Willart J.-F., Danede F., Lacante E., Vincent C., Siepmann F., Siepmann J. (2016). Ear cubes for local controlled drug delivery to the inner ear. Int. J. Pharm..

[b0045] Gehrke M., Verin J., Gnansia D., Tourrel G., Risoud M., Vincent C., Siepmann F., Siepmann J. (2019). Hybrid ear cubes for local controlled dexamethasone delivery to the inner ear. Eur. J. Pharm. Sci..

[b0050] Hahn H., Salt A.N., Biegner T., Kammerer B., Delabar U., Hartsock J.J., Plontke S.K. (2012). Dexamethasone levels and base-to-apex concentration gradients in the scala tympani perilymph after intracochlear delivery in the guinea pig. Otol. Neurotol..

[b0055] Jia H., François F., Bourien J., Eybalin M., Lloyd R.V., Van De Water T.R., Puel J.L., Venail F. (2016). Prevention of trauma-induced cochlear fibrosis using intracochlear application of anti-inflammatory and antiproliferative drugs. Neuroscience.

[b0060] Krenzlin S., Vincent C., Munzke L., Gnansia D., Siepmann J., Siepmann F. (2012). Predictability of drug release from cochlear implants. J. Control. Release.

[b0065] Liu Y., Jolly C., Braun S., Janssen T., Scherer E., Steinhoff J., Ebenhoch H., Lohner A., Stark T., Kiefer J. (2015). Effects of a dexamethasone-releasing implant on cochleae: a functional, morphological and pharmacokinetic study. Hear. Res..

[b0070] Mäder K., Lehner E., Liebau A., Plontke S.K. (2018). Controlled drug release to the inner ear: concepts, materials, mechanisms, and performance. Hear. Res..

[b0075] Plontke S.K., Glien A., Rahne T., Mäder K., Salt A.N. (2014). Controlled release dexamethasone implants in the round window niche for salvage treatment of idiopathic sudden sensorineural hearing loss. Otol. Neurotol..

[b0080] Plontke S.K., Götze G., Rahne T., Liebau A. (2017). Intracochlear drug delivery in combination with cochlear implants: current aspects. HNO.

[b0085] Plontke S.K., Mikulec A., Salt A.N. (2007). Rapid clearance of methylprednisolone after intratympanic application in humans. Comment on: Bird PA, Begg EJ, Zhang M, et al. Intratympanic versus intravenous delivery of methylprednisolone to cochlear perilymph. Otol. Neurotol..

[b0090] Plontke S.K., Salt A.N. (2018). Local drug delivery to the inner ear: principles, practice, and future challenges. Hear. Res..

[b0095] Plontke S.K., Siedow N., Wegener R., Zenner H.-P., Salt A.N. (2007). Cochlear pharmacokinetics with local inner ear drug delivery using a three-dimensional finite-element computer model. Audiol. Neurootol..

[b0100] Roy R., Hedge S. (2013). Split Ozurdex implant: a caution. Can. J. Ophthalmol..

[b0105] Salt A.N. (2002). Simulation of methods for drug delivery to the cochlear fluids. Adv. Otorhinolaryngol..

[b0110] Salt A.N., Hartsock J.J., Gill R. (2012). Perilymph pharmacokinetics of markers and dexamethasone applied and sampled at the lateral semi-circular canal. J. Assoc. Res. Otolaryngol..

[b0115] Salt A.N., Plontke S.K. (2018). Pharmacokinetic principles in the inner ear: influence of drug properties on intratympanic applications. Hear. Res..

[b0120] Salt A.N., Plontke S.K. (2009). Principles of local drug delivery to the inner ear. Audiol. Neurootol..

[b0125] Skinner M.W., Holden T.A., Whiting B.R., Voie A.H., Brunsden B., Neely J.G., Saxon E.A., Hullar T.E., Finley C.C. (2007). In vivo estimates of the position of advanced bionics electrode arrays in the human cochlea. Ann. Otol. Rhinol. Laryngol. Suppl..

[b0130] Takumi Y., Nishio S.Y., Mugridge K., Oguchi T., Hashimoto S., Suzuki N., Iwasaki S., Jolly C., Usami S.I. (2014). Gene expression pattern after insertion of dexamethasone-eluting electrode into the guinea pig cochlea. PLoS One.

[b0135] Wilk M., Hessler R., Mugridge K., Jolly C., Fehr M., Lenarz T., Scheper V. (2016). Impedance changes and fibrous tissue growth after cochlear implantation are correlated and can be reduced using a dexamethasone eluting electrode. PLoS One.

